# Species identification of *Enterococcus* spp: Whole genome sequencing compared to three biochemical test‐based systems and two Matrix‐Assisted Laser Desorption/Ionization Time‐of‐Flight Mass Spectrometry (MALDI‐TOF MS) systems

**DOI:** 10.1002/jcla.23348

**Published:** 2020-05-01

**Authors:** Elvira Garza‐González, Paola Bocanegra‐Ibarias, An Dinh, Rayo Morfín‐Otero, Adrián Camacho‐Ortiz, Fabián Rojas‐Larios, Patricia Rodríguez‐Zulueta, Cesar A. Arias

**Affiliations:** ^1^ Hospital Universitario “Dr. José Eleuterio González” Universidad Autónoma de Nuevo León Monterrey Mexico; ^2^ Division of Infectious Diseases and Center for Antimicrobial Resistance and Microbial Genomics (CARMiG) McGovern Medical School UTHealth Houston TX USA; ^3^ Centro Universitario de Ciencias de la Salud Hospital Civil de Guadalajara “Fray Antonio Alcalde” e Instituto de Patología Infecciosa y Experimental Universidad de Guadalajara Guadalajara Mexico; ^4^ Facultad de Medicina Universidad de Colima Colima Mexico; ^5^ Hospital General Dr. Manuel Gea González Mexico City Mexico; ^6^ School of Public Health Center for Infectious Diseases UTHealth Houston TX USA; ^7^ Molecular Genetics and Antimicrobial Resistance Unit and International Center for Microbial Genomics Universidad El Bosque Bogotá Colombia

**Keywords:** MALDI TOF, phoenix, Vitek MS, whole genome sequenicng

## Abstract

**Aim:**

Here, we evaluated the performance of two commercial MALDI‐TOF MS systems and three biochemical‐based systems and compared them to WGS as the gold standard for identifying isolates of vancomycin‐resistant enterococci (VRE).

**Methods:**

A total of 87 VRE clinical isolates were included. The mass spectrometers were the Microflex system with Biotyper software 3.1 and the Vitek MS system. The biochemical‐based systems included the Vitek 2, Phoenix, and MicroScan WalkAway systems. WGS was performed on an Illumina MiSeq instrument using the MiSeq v3 reagent kit. Vancomycin resistance was determined according to CLSI criteria.

**Results:**

Among the 87 VRE, 71 and 16 were identified as *Enterococcus faecium* and *Enterococcus faecalis* by WGS. All 71 *E faecium* were correctly identified by both mass spectrometers, as well as the Vitek 2 and Phoenix instruments. However, only 51 *E faecium* isolates were correctly identified by the MicroScan system. The most frequent misidentification was *Enterococcus casseliflavus* (n = 20). For vancomycin‐resistant *E faecium*, the Microflex Biotyper system had the highest sensitivity (85.54%), and all instruments (except for the Microscan) had a 100% specificity and PPV. Up to 87% of *E faecalis* isolates were misidentified by VITEK MS and VITEK2, 81% by Microscan and Phoenix, and 75% by Bruker biotyper.

**Conclusion:**

As the coverage of type strain‐genome sequence database continues to grow and the cost of DNA sequencing continues to decrease, genome‐based identification can be a useful tool for diagnostic laboratories, with its superior accuracy even over MALDI‐TOF and database‐driven operations.

## 1 INTRODUCTION

Biochemical test‐based methodologies are still used in some laboratories for the identification of bacterial isolates, mainly because of the need for the performance of drug susceptibility tests, to identify bacterial isolates.^1^ The biochemical‐based systems include the Vitek 2 (bioMérieux), Phoenix (Becton‐Dickinson), and MicroScan WalkAway (Siemens Healthcare Diagnostics).^2^ However, these systems are unable to distinguish some species, especially among Gram‐positive bacteria.^3^, ^4^

Matrix‐assisted laser desorption ionization time‐of‐flight mass spectrometry (MALDI‐TOF MS) is an efficient method used in clinical microbiology laboratories for the identification of clinically relevant microorganisms. Microorganisms can be identified using a reference database in a turnaround time of minutes, which is essential when rapid results are needed.^5^, ^6^, ^7^ At present, MALDI‐TOF MS has been adopted for the identification of pathogenic bacteria.^8^

Because whole‐genome sequencing (WGS) costs are continuously decreasing, its use as part of the routine clinical microbiology laboratory is starting to be used in large hospitals.^9^

Here, we evaluated the performance of two commercial MALDI‐TOF MS systems and three biochemical‐based systems and compared them to WGS as the gold standard for identifying isolates of vancomycin‐resistant enterococci (VRE), using a use an in‐silico MLST method for species identification and ST calls.

## 2 METHODS

A total of 87 VRE clinical isolates recovered from urine (33%), soft tissue (24%), blood (16%), and other specimens (27%) were identified by two commercial MALDI‐TOF MS and three biochemical‐based systems.

For both MALDI‐TOF instruments, the direct method was used for identification. The mass spectrometers were the Microflex system with Biotyper software 3.1 (Biotyper; Bruker Daltonics) and the Vitek MS system (BioMérieux) with the software MYLA ver 4.6.1. Both instruments were used according to the manufacturer's instructions.

The biochemical‐based systems included the Vitek 2 (bioMérieux), Phoenix (Becton‐Dickinson), and MicroScan WalkAway (Siemens Healthcare Diagnostics) systems. All systems were used according to the manufacturer's instructions.

Vancomycin susceptibility was determined using the broth microdilution method according to the 2019 Clinical and Laboratory Standards Institute guidelines and breakpoint criteria in the document M100‐S29.^10^

For WGS, DNA from the 87 bacterial overnight cultures was isolated using a QIAamp DNA mini kit (Qiagen). Genomic libraries were prepared using the NexteraXT kit (Illumina, Inc.) according to the manufacturer's instructions, and a 300‐bp paired‐end sequencing run was performed on an Illumina MiSeq instrument using the MiSeq v3 reagent kit (Illumina Inc.). Reads for each isolate were assembled de novo with SPAdes v3.11.1,^11^ and mlst v2.10 (https://github.com/tseemann/mlst) was used for in‐silico multilocus sequence typing against the PubMLST database.

The sensitivity and specificity, and the positive and negative predictive values for each instrument, were determined using the GraphPad Prism 6 software.

## 3 RESULTS

Among the 87 VRE, 71 and 16 were identified as *E faecium* and *E faecalis* by WGS. All 71 *E faecium* were correctly identified by both mass spectrometers, as well as the Vitek 2 and Phoenix instruments. However, only 51 *E faecium* isolates were correctly identified by the MicroScan system (Table 1). The most frequent misidentification was *E casseliflavus* (n = 20).

**Table 1:** Distribution of species identification among instruments used

WGS	Bruker Biotyper	Vitek MS	Vitek 2	MicroScan	Phoenix
*E faecium* (n = 71)	*E faecium* (n = 71)	*E faecium* (n = 71)	*E faecium* (n = 71)	*E faecium* (n = 51)	*E faecium* (n = 71)
				*E casseliflavus (*n* = 20)*	
*E faecalis* (n = 16)	*E faecalis* (n = 4)	*E faecalis* (n = 2)	*E faecalis (*n* = 2)*	*E faecalis* (n = 3)	*E faecalis* (n = 3)
	*E faecium* (n = 12)	*E faecium* (n = 14)	*E faecium* (n = 14)	*E faecium* (n = 10)	
				*E casseliflavus* (n = 3)	*E faecium* (n = 13)

Abbreviations: *E casseliflavus*, *Enterococcus faecium*; *E faecalis*, *Enterococcus faecalis*; *E faecium*, *Enterococcus faecium*; WGS, whole genome sequencing.

For vancomycin‐resistant *E faecium*, the Microflex Biotyper system had the highest sensitivity (85.54%), and all instruments (except for the Microscan) had a 100% specificity and PPV. The NPV was lower than 38% for all devices. The instrument with the highest accuracy was the Bruker Biotyper system (86.21%) (Table 2).

**Table 2:** Sensitivity, specificity, negative predictive value and positive predictive value for each instrument when identifying *Enterococcus faecium*

Test, % (95%CI)	Bruker biotyper	Vitek MS	Vitek 2	Microscan	Phoenix
Sensitivity	85.54 (76.11‐92.30)	83.53 (73.91‐(90.69	83.53 (73.91‐90.69)	83.61 (71.9‐91.85)	84.52 (74.99‐91.49)
Specificity	100.00 (39.76‐100.00)	100.00 (15.81‐100.00)	100.00 (15.81‐100.00)	23.08 (8.97‐43.65)	100.00 (29.24‐100.00)
PPV	100.00	100.00	100.00	71.83 (66.78‐76.39)	100.00
NPV	25.00 (16.49‐36.00)	12.50 (8.13‐18.74)	12.50 (8.13‐18.74)	37.50 (19.58‐59.66)	18.75 (12.28‐27.56)
Accuracy	86.21 (77.15‐92.66)	83.91(74.48‐90.91)	83.91 (74.48‐90.91)	65.52 (54.56‐75.39)	85.06 (75.80‐91.80)

Abbreviations: NPV, negative predictive value; PPV, positive predictive value.

For *E faecalis*, only four isolates were correctly identified by the Bruker biotyper and two by the Vitek MS (Table 2). Up to 87% of *E faecalis* isolates were misidentified by VITEK MS and VITEK2, 81% by Microscan and Phoenix, and 75% by Bruker biotyper.

## 4 DISCUSSION

MALDI‐TOF MS has shorter turnaround times and lower costs versus automated instruments utilizing biochemical tests. MALDI‐TOF MS identifies isolates by comparing their proteomic profiles to reference strains in protein databases; thus, the accuracy is dependent on the software and spectral database/libraries.^8^ By contrast, species identification by WGS depends on deposited genomes and is less likely to have errors due to media and growth conditions. If reference genome sequences for bacterial strain types are available, any isolate could be identified with high confidence.^12^

Unrelated but genetically similar vancomycin‐resistant *Enterococcus* spp. (including *E casseliflavus*, (which have intrinsic resistance to vancomycin) may be isolated as causative agents of infection, so typing methods are needed to determine the genetic relatedness of isolates. Previous studies have demonstrated the superiority of WGS over pulse‐field gel electrophoresis and multi‐locus sequence typing to study the strain relatedness of vancomycin‐resistant Enterococci^13^, ^14^, ^15^).

We decided to use the WGS as a gold standard because WGS overcomes the limitations of MLST and other techniques, including the sequencing and analysis of the 16S rRNA gene. Sequencing of the 16S rRNA gene is used for the identification of bacteria but has limitations for some bacterial groups, including *Enterococcus* spp, because of the high degree of identity of this gene.^16^ Furthermore, MLST is not ideal for identification of enterococci, due to high rates of allele variation in enterococci.

The core genome of *E faecium* has been defined in many ways, but we have used cutting edge definitions since *E faecium* is a species that evolves through recombination.^17^ Thus, we are confident that core genome analyses are accurate.

As the coverage of type strain‐genome sequence database continues to grow and the cost of DNA sequencing continues to decrease, genome‐based identification can be a useful tool for diagnostic laboratories, with its superior accuracy even over MALDI‐TOF and database‐driven operations.

## ETHICAL APPROVAL

This protocol was approved by the Ethical Committee in Investigation of the Hospital Civil de Guadalajara ‘Fray Antonio Alcalde’ (Reg no. 121/17).
